# Clinical manifestations, serotype distribution, and incidence of pediatric invasive pneumococcal disease in Catalonia (Spain), 2018–2022

**DOI:** 10.1007/s00431-025-06137-1

**Published:** 2025-05-02

**Authors:** Mariona F. de Sevilla, Claudia Alcaraz-Soler, Nuria Soldevila, Conchita Izquierdo, Cristina Esteva, Fernando Moraga-Llop, Sebastià González-Peris, Pilar Ciruela, Alvaro Díaz-Conradi, Amaresh Pérez-Argüello, Belén Viñado, Angela Domínguez, Juan José García-García, Carmen Muñoz-Almagro

**Affiliations:** 1 Pediatric Department, Hospital Sant Joan de Déu Barcelona, Passeig Sant Joan de Déu Number 2, 08950 Esplugues de Llobregat, Barcelona Spain; 2 Institut de Recerca Sant Joan de Déu, Barcelona, Spain; 3 Consorcio de Investigación Biomédica en Red de Epidemiología y Salud Pública (CIBERESP), Madrid, Spain; 4 Universitat de Barcelona, Barcelona, Spain; 5 Agència de Salut Pública de Catalunya, Generalitat de Catalunya, Barcelona, Spain; 6 Microbiology Department, Hospital Sant Joan de Déu Barcelona, Barcelona, Spain; 7 Pediatric Department, Hospital Vall d’Hebron, Barcelona, Spain; 8 Pediatric Department, Hospital HM Nens, Barcelona, Spain; 9 Microbiology Department, Hospital Vall d’Hebron, Barcelona, Spain; 10 Universitat Internacional de Catalunya, Barcelona, Spain

**Keywords:** Invasive pneumococcal disease, Serotype, Serotype 3, Pneumonia, Pneumococcal conjugate vaccine, PCV13, PCV15, PCV20, Vaccine failure

## Abstract

The global incidence of invasive pneumococcal disease (IPD) decreased after the switch from PCV7 to PCV13 in 2010. However, serotype 3 remains the leading cause of IPD in Catalonia (Spain), due to the low effectiveness of PCV13 against it. This study aimed to analyze the clinical, epidemiological, and microbiological characteristics of IPD in children over 5 years and evaluate the potential impact of new vaccines (PCV15 and PCV20). A 5-year prospective observational study was conducted from 2018 to 2022, including children up to 18 hospitalized with IPD at three major children’s hospitals in Catalonia. Data on clinical, epidemiological, and microbiological factors were collected. A total of 220 episodes were identified, with a median age of 33.0 months (range 0–209). Comparing pre-pandemic (2018–2019) to early pandemic years (2020–2021), the IPD rate in children < 18 years decreased by 60.6% (*p* < 0.001). However, no significant change was observed when comparing 2022 to 2018. The most common diagnoses were pneumonia (61.8%), meningitis (14.5%), and bacteremia without focus (13.2%). Serotype 3 was the leading cause (35.1%) of IPD and was associated with complicated pneumonia (84.7%) and vaccine failure (73.6%). Ninety-three IPD episodes (45.4%) were caused by PCV13 serotypes, 97 (47.3%) by PCV15 serotypes, and 132 (64.4%) by PCV20 serotypes. *Conclusion*: The incidence of IPD has remained stable, except for a decrease during the pandemic. Serotype 3 was the most common, often associated with vaccine failures and severe pneumonia. PCV15 and PCV20 vaccines could offer better coverage against circulating serotypes and further reduce IPD incidence in Catalonia.

**Table:** 

**What is Known:** *• Serotype 3 remains a leading cause of invasive pneumococcal disease (IPD) despite inclusion in PCV13 due to its limited vaccine effectiveness.* *• IPD incidence decreased globally during the COVID-19 pandemic, likely due to public health measures.*	
**What is New:** *• In Catalonia, serotype 3 continues to dominate pediatric IPD cases and is frequently associated with complicated pneumonia and vaccine failure.* *• PCV15 and PCV20 offer broader serotype coverage and may significantly improve IPD prevention in children.*

**What is Known:**

*• Serotype 3 remains a leading cause of invasive pneumococcal disease (IPD) despite inclusion in PCV13 due to its limited vaccine effectiveness.*

*• IPD incidence decreased globally during the COVID-19 pandemic, likely due to public health measures.*

**What is New:**

*• In Catalonia, serotype 3 continues to dominate pediatric IPD cases and is frequently associated with complicated pneumonia and vaccine failure.*

*• PCV15 and PCV20 offer broader serotype coverage and may significantly improve IPD prevention in children.*

## Introduction

*Streptococcus pneumoniae* is a common cause of invasive infection in children, such as community-acquired pneumonia, meningitis, and bacteremia, and it poses a significant health burden globally [1]. In Europe, a confirmed case of invasive pneumococcal disease (IPD) is defined as the presence of clinical findings of infection together with isolation and/or DNA detection of *S. pneumoniae* in normal sterile body fluid such as blood, cerebrospinal fluid, pleural fluid, or articular fluid https://www.ecdc.europa.eu/en/all-topics/eu-case-definitions. *Streptococcus pneumoniae* is the leading cause of death worldwide for children under the age of 5, and it is associated with more than 40 million years of life lost [1, 2].

Over time, more than one hundred serotypes of *Streptococcus pneumoniae* have been discovered [3]. Vaccination with pneumococcal conjugate vaccines has successfully reduced infections caused by the targeted pneumococcal serotypes [2, 4]. According to the WHO data from 2018, deaths caused by pneumococcal disease decreased by 51% after the introduction of conjugate vaccines between 2000 and 2015 [1].

Spain first introduced the 7-valent pneumococcal conjugate vaccine (PCV7) for children in 2001. This vaccine targeted seven serotypes (4, 6B, 9 V, 14, 18 C, 19 F, and 23 F) but was publicly funded only for children at high risk of invasive pneumococcal disease (IPD). All other children had to purchase it privately. PCV7 effectively reduced infections caused by the included serotypes, but over time, infections caused by non-vaccine serotypes increased [5–7].

To address this problem, the 13-valent pneumococcal conjugate vaccine (PCV13) was introduced in June 2010, also available only through the private market [4, 5]. PCV13 included additional serotypes (1, 3, 5, 6 A, 7 F, and 19 A) to address the increasing cases of IPD caused by non-PCV7 serotypes. After the switch from PCV7 to PCV13, the overall incidence of pediatric IPD decreased significantly, particularly for serotypes 1 and 19 A [1, 5, 8, 9]. However, the benefits of PCV13 were somewhat reduced due to serotype substitution and lower efficacy against serotype 3 [8–10].

In July 2016, systematic vaccination with PCV13 was introduced for all children in Catalonia using the 2 + 1 schedule: two doses in early infancy (at 2 and 4 months), followed by a booster at 11 months.

**Fig. 1:**
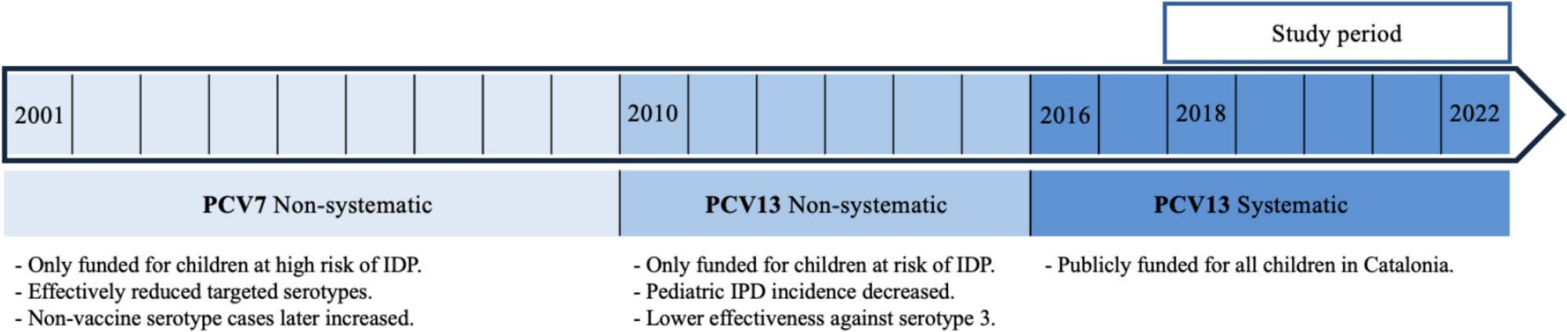
Successive introduction of different pneumococcal conjugate vaccines in the pediatric population in Catalonia (Spain)

Figure 1 provides a visual representation of the introduction of pneumococcal conjugate vaccines (PCVs) in the pediatric population of Catalonia, Spain, showing which vaccines were available at different times and their main impact.

Regarding the evolution of the IPD in recent years, our group reported a preliminary warning describing how the COVID- 19 pandemic radically changed the epidemiology of IPD in Catalonia, with a dramatic decrease in the disease in 2020 [11], as happened all over the world [12]. Now we want to complete our preliminary report by analyzing whether a re-emergence of IPD is taking place in our environment [13, 14].

This study focuses on the pediatric population of Catalonia. The PCV13 vaccine was consistently included in the systematic vaccination schedule for the pediatric population throughout the study period (2018–2022). We aim to describe the clinical, epidemiological, and microbiological characteristics of IPD in the pediatric population of Catalonia before the significant change that occurred with the introduction of new conjugate vaccines available for children in 2023: PCV15 and PCV20.

The immunogenicity of the 15-valent vaccine has proven superior to PCV13 for serotype 3 and the two new serotypes (22 F and 33 F) and non-inferior for the remaining 12 shared serotypes. In addition, it has a successful safety profile for children and adults [15–17]. In October 2023, the Catalonian health authorities replaced PCV13 with PCV15 in the routine pediatric vaccination schedule, with doses scheduled at 2, 4, and 11 months of age. Additionally, since 2023, the PCV20 vaccine has been included in the systematic vaccination schedule for the adult population older than 65 years old, replacing the 23-valent polysaccharide vaccine [18, 19].

The results of this study will be used to evaluate the potential impact of these new pneumococcal vaccines.

## Materials and methods

### Patients

We performed a prospective study comprising all children < 18 years with IPD managed at the three leading children’s hospitals of Catalonia, Spain (Hospital Sant Joan de Deu, Hospital Vall d’Hebron, and HM Nens), during a 5-year period (January 2018–December 2022). In 2022, the total number of children’s hospitalizations in Catalonia was 91,659. Hospital Sant Joan de Deu captured 19,605 hospitalizations (21.4%), Hospital Vall d’Hebron 8061 (8.8%), and Hospital HM Nens 4970 (5.4%). During that year, these hospitals served a pediatric population of 489,314 children < 18 years (35.6% of the Catalan pediatric population < 18 years old) [20, 21].

### Definitions

An episode of IPD was defined as the presence of clinical findings of infection together with isolation and/or DNA detection of lytA gene and an additional capsular gene of *S. pneumoniae* by real-time PCR in any sterile body fluid such as blood, cerebrospinal fluid, pleural fluid, articular fluid, or mastoidal sample https://www.ecdc.europa.eu/en/all-topics/eu-case-definitions [22, 23].

Pneumonia was categorized into two types: uncomplicated and complicated. Complicated pneumonia included pleural effusion, empyema, and necrotizing pneumonia. Uncomplicated pneumonia was considered one where there is pulmonary consolidation without the presence of complications such as pleural effusion or signs of necrosis, such as pneumothorax or pneumatoceles.

### Data confidentiality and ethical aspects

No diagnostic tests or samples were taken from any participant besides those required by routine care. The study complies with the principles of the Declaration of Helsinki and the legal structure in respect to international human rights and biomedicine and protection of personal data laws. The study was approved by the Ethics Committee of Fundació Sant Joan de Déu (PIC- 157–17). Informed consent signed by parents or legal guardians was given to all participants. All data were treated as confidential, and records were accessed anonymously.

### Data collected and analyzed

Epidemiologic characteristics included age, gender, underlying medical condition, and immunization status against *S. pneumoniae*. Vaccination status was obtained by personal vaccination card or electronic primary care information. PCV13 vaccine failure was defined, according to the Council for the International Organizations of Medical Sciences and the WHO Working Group, as the occurrence of the illness in a person who was appropriately and fully vaccinated [24]. Point out that at least 15 days must pass since the last dose received in order to be considered a vaccination failure.

We analyzed factors that increase the risk for pneumococcal disease, including chronic medical conditions (such as cyanotic congenital heart disease, congenital or acquired humoral immunodeficiency, human immunodeficiency virus infection, absent or deficient splenic function, abnormal innate immune responses, cochlear implants, or cerebrospinal fluid [CSF] leak) [25].

Clinical characteristics include clinical presentation (pneumonia, meningitis, bacteremia without focus, osteoarticular infection, mastoiditis or sepsis), need for admission to the intensive care unit (ICU), complications, antibiotic treatment and duration, days of hospitalization, and clinical outcome.

Microbiological data were also recorded, including *S. pneumoniae* serotype, clonal type, and antimicrobial susceptibility to penicillin/cefotaxime.

### Microbiological methods

All pneumococcal isolates were first identified by optochin sensitivity test and an antigenic test targeting the capsular polysaccharide (Slidex pneumo-kit, BioMérieux, Marcy-l’Etolie, France). Detection of *S. pneumoniae* DNA was performed by real-time PCR according to published assays, which include the amplification of the lytA gene and an additional capsular gene [22]. Molecular methods, including whole genome sequencing analysis, performed the capsular typing of strains according to previously published methods [26, 27]. When the diagnosis was given only by real-time PCR, the detection of pneumococcal serotypes was performed following a previously validated molecular method which depends on the bacterial load obtained by real-time PCR (Table 1) [23, 28].

**Table 1:** Detection of pneumococcal serotypes by real-time PCR

Ct value	Additional capsular gene detected	Serotypes identified
** < 30** (high DNA bacterial load)	cpsA/wzg	1, 2, 3, 4, 5, 6 A/6B, 6 C, 7 F/7 A, 7 C/7B/40, 8, 9 V/9 A, 9 N/9L, 10 A, 10 F/10 C/33 C, 11 A/11D, 12 F/12 A/44/46, 13, 14, 15 A/15 F, 15B/15 C, 16 F, 17 F, 18 A/18B/18 C/18D, 19 A, 19 F, 20, 21, 22 F/22 A, 23 A, 23B, 23 F, 24 A/24B/24 F, 31, 33 F/33 A/37, 34, 35 A, 35 C/42, 35B, 35 F/47 F, 38/25 F
** ≥ 30** (low DNA bacterial load)	cpsA/wzg	1, 2, 3, 4, 5, 6 A, 6B, 6 C/6D, 7 F/7 A, 8, 9 V/9 A/9 N/9L, 11 A, 11D, 12 F/12 A/12B/44/46, 14, 15 A/15 F, 16 F, 18 C/18B, 19 A, 19 F/19B/19 C, 22 F/22 A, 23 A, 23 F, 33 F/33 A/37

Susceptibility to penicillin and third-generation cephalosporin was defined according to EUCAST ECOFF breakpoints (meningeal breakpoints). Non-susceptibility to penicillin was considered when MIC ≥ 0.06 µg/mL [29]. We decided to use meningeal breakpoints for all invasive infections because they represent the most conservative and, therefore, safest values for interpreting the effectiveness of treatment. Furthermore, meningeal breakpoints, which coincide with epidemiological cut-off values for *S. pneumoniae*, are recommended in epidemiological surveillance studies [29].

Clonal composition of strains was analyzed using multilocus sequence typing (MLST) by Sanger Sequencing until 2019 and Whole Genome Sequencing since 2020 [26]. The assignment of alleles and sequence types (ST) was done using the software at the pneumococcal web page www.mlst.net and the platform pathogenwatch web page https://pathogen.watch/. Analysis of ST and assignment to clonal complex (CC) was performed with the eBURST program [30]. ST that shared six of seven alleles (single locus variants [SLV]) were considered a clonal complex.

### Statistical analysis

*Χ*^2^ test or Fisher’s exact test was used to compare proportions. Since the data do not follow a normal distribution, all parameters are reported as medians, and the median test, a non-parametric test used to compare medians, was applied. Statistical analyses were performed using SPSS for Windows, version 29 (SPSS), and Epi Info, version 3.01 (Centres for Disease Control and Prevention). We calculated 95% CIs, and 2-sided *p* values ≤ 0.05 were statistically significant.

## Results

During the study period, 220 episodes of IPD were identified, including 111 male patients (50.5%) and 109 female patients (49.5%), with a median age of 33.0 months (range 0–209). A total of 146 episodes (66.4%) were in children ≥ 24 months, 62 (28.2%) in children 7–23 months, and 12 (5.4%) in children ≤ 6 months of age.

According to the criteria of the American Academy of Pediatrics, 27 of 220 (12.3%) children were at high risk of IPD, including 12 children with malignant disease who were actively receiving immunosuppressive therapy, 5 with cyanotic congenital cardiopathy, 3 with cerebrospinal fluid fistula, 2 with chronic renal failure, 2 with cochlear implants, and 3 with congenital immunodeficiency.

Regarding PCV13 immunization, 140 (64.2%) episodes occurred in children who were fully vaccinated, 52 (23.9%) episodes occurred in children who were partially vaccinated, and 26 (11.9%) episodes occurred in children who were not vaccinated. In two episodes, the vaccination status was unknown.

### Clinical presentation

Table 2 summarizes the clinical diagnoses of the patients enrolled in this study.

**Table 2:** Clinical diagnoses

Clinical presentation	Number of episodes (*n*)	Percentage (%)
**Pneumonia**	**136**	**61.8% of episodes**
Uncomplicated pneumonia	31	22.8% of pneumonia episodes
Complicated pneumonia	105	77.2% of pneumonia episodes
Empyema	45	42.9% of complicated pneumonia
Empyema + Necrotizing pneumonia	42	40% of complicated pneumonia
Parapneumonic pleural effusion	10	9.5% of complicated pneumonia
Parapneumonic pleural effusion + necrotizing pneumonia	8	7.6% of complicated pneumonia
**Meningitis**	**32**	**14.5% of episodes**
**Bacteremia without focus**	**29**	**13.2% of episodes**
**Osteoarticular infection**	**10**	**4.6% of episodes**
**Sepsis**	**6**	**2.7% of episodes**
**Mastoiditis**	**6**	**2.7% of episodes**
**Endophthalmitis**	**1**	**0.5% of episodes**

All the children were admitted to the hospital. The median length of stay was 11 days (range 0–89). The most prolonged median stay by clinical presentation was 16 days (range 11–78) for patients with sepsis.

Sixty-eight episodes (31.0%) required admission to the pediatric intensive care unit (PICU). Overall, 24 of 32 (75%) episodes of meningitis were admitted to PICU, 4 of 6 (66.7%) episodes of sepsis, and 3 of 6 (50%) episodes of mastoiditis. Most meningitis patients who did not require PICU admission were previously healthy children in generally good condition.

Among the 220 reported cases, one fatality occurred: a 6-year-old female who had contracted meningitis and was partially vaccinated with two doses of PCV13 (at 2 and 4 months old), being the identified serotype 12 F. A total of 175 (79.5%) episodes were cured at discharge, and 44 (20%) were discharged with sequelae, mainly neurological, in the case of meningitis, and respiratory, in the case of complicated pneumonia.

Table 3 shows the epidemiological and clinical characteristics of patients with IPD.

**Table 3:** Epidemiological and clinical characteristics of 220 patients with IPD

Clinical presentation	Number of episodes	Gender	Age	Underlying condition	Length of stay in days (median, range)	PICU admission	Length of stay in PICU (median, range)	Days of antibiotic (median, range)
Complicated pneumonia	105	50 (47.6%) female 55 (52.4%) male	0 (0%) ≤ 6 months 22 (21.0%) 7–23 months 83 (79.0%) ≥ 24 months	2 (1.9%)	12 (2–89)	29 (27.6%)	6 (1–66)	21 (7–83)
Uncomplicated pneumonia	31	19 (61.3%) female 12 (38.7%) male	1 (3.2%) ≤ 6 months 14 (45.2%) 7–23 months 16 (51.6%) ≥ 24 months	5 (16.1%)	6 (2–25)	6 (19.4%)	4 (2–17)	10 (1–23)
Meningitis	32	16 (50.0%) female 16 (50.0%) male	7 (21.9%) ≤ 6 months 7 (21.9%) 7–23 months 18 (56.3%) ≥ 24 months	5 (15.6%)	15 (0–55)	24 (75.0%)	4 (1–23)	14 (2–66)
Bacteremia	29	14 (48.2%) female 15 (51.7%) male	3 (10.3%) ≤ 6 months 11 (37.9%) 7–23 months 15 (51.7%) ≥ 24 months	10 (34.4%)	6 (0–56)	2 (6.8%)	7 (1–20)	8 (5–21)
Osteoarticular infection	10	5 (50.0%) female 5 (50.0%) male	0 (0%) ≤ 6 months 7 (70%) 7–23 months 3 (30%) ≥ 24 months	1 (10.0%)	6 (3–30)	0 (0%)	-	26 (15–38)
Mastoiditis	6	2 (33.3%) female 4 (66.7%) male	0 (0%) ≤ 6 months 1 (16.7%) 7–23 months 5 (83.3%) ≥ 24 months	0 (0%)	12 (6–69)	3 (50.0%)	34 (1–4)	26 (4–69)
Sepsis	6	2 (33.3%) female 4 (66.7%) male	1 (16.7%) ≤ 6 months 0 (0%) 7–23 months 5 (83.3%) ≥ 24 months	4 (66.7%)	16 (11–78)	4 (66.7%)	4 (1–13)	15 (7–39)
Endophthalmitis	1	1 (100%) female	1 (100%) ≥ 24 months	0 (0%)	16	0 (0%)	-	24

### Incidence

**Fig. 2:**
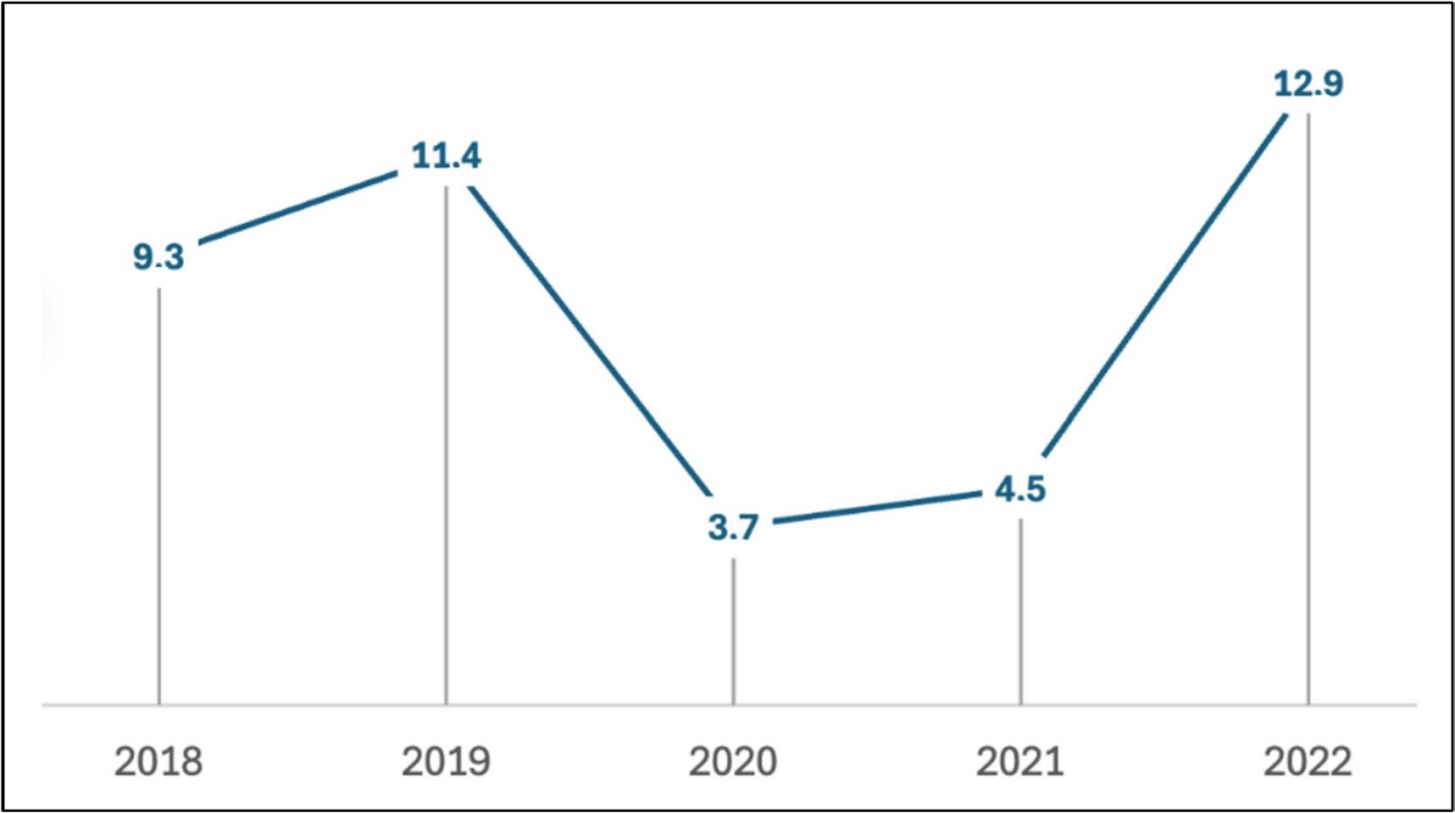
Incidence rate per 100,000 inhabitants < 18 years old

Significant changes in IPD incidence rates were observed during the study period, as shown in Fig. 2.

Comparing the rate of pre-pandemic years (2018–2019) vs early pandemic years (2020–2021), the rate of IPD in children < 18 years decreased from 10.4 episodes per 100,000 population to 4.1 episodes per 100,000 population (a decrease of 60.6%; 95% confidence interval, 43.8%; 72.0%) *p* < 0.001. However, comparing the last year of the study (2022) vs the first (2018), non-significant differences were observed: 12.9 episodes vs 9.3 episodes per 100,000 population (an increase of 38.7; 95% confidence interval, − 4.3%; 98.7%) *p* = 0.05.

There was a notable seasonal variation: 65.5% of episodes were detected during cool months (October to March) versus 34.5% during warm months (April to September).

### Serotypes, molecular study, and antibiotic susceptibility

The IPD diagnosis was established in 51 episodes (23.2%) by bacterial culture and real-time PCR, 55 (25.0%) episodes only by culture, and 114 (51.8%) only by real-time PCR.

Serotyping was performed in 205 (93.2%) of the 220 IPD episodes (15 patients had insufficient sample for capsular typing or the strain was not viable).

**Fig. 3:**
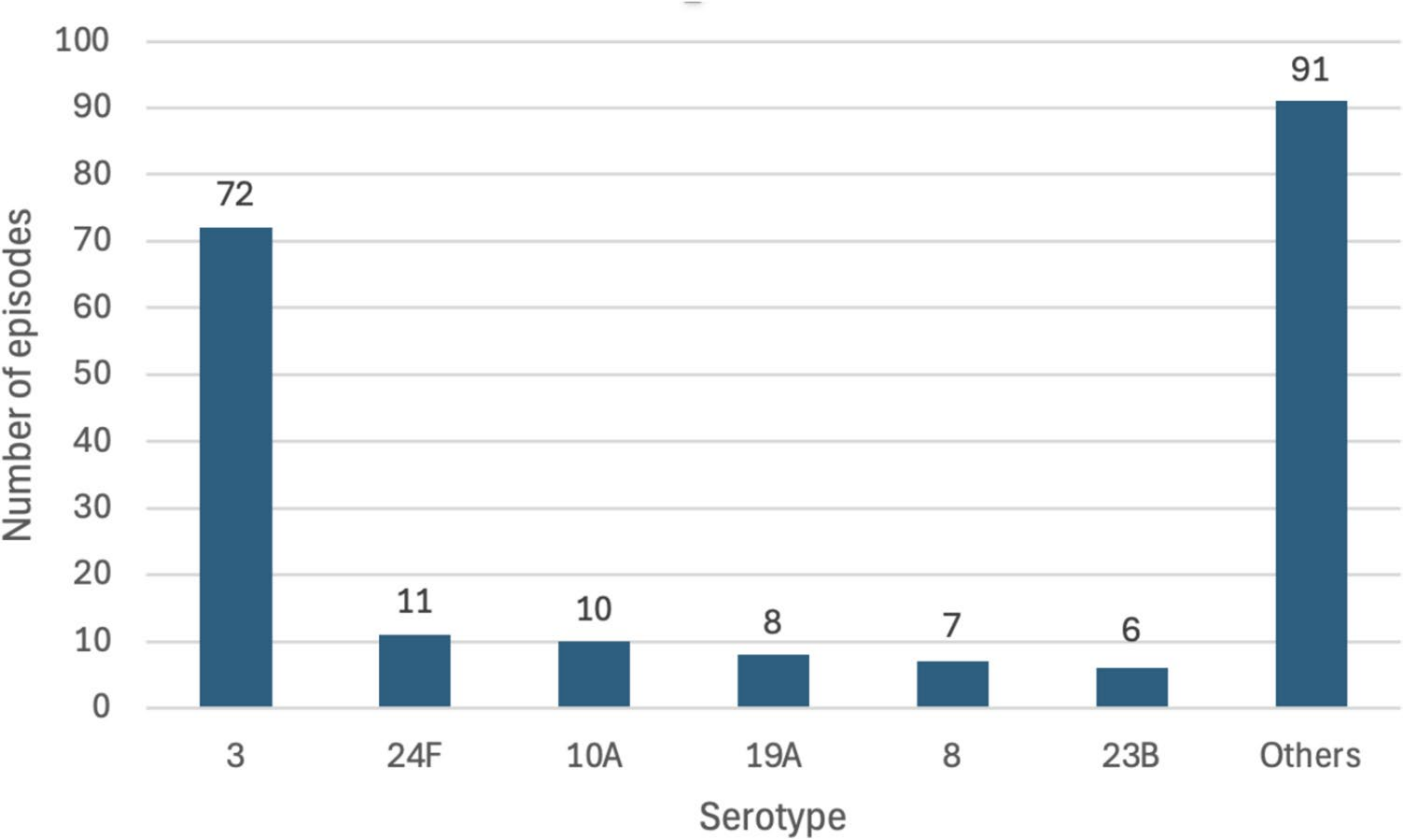
Serotype distribution during the study period 2018–2022

The most frequent among identified serotypes was serotype 3 (72; 35.1%). Other frequent serotypes were 24 F (11; 5.4%), 10 A (10; 4.9%), 19 A (8; 3.9%), 8 (7; 3.4%), and 23 B (6; 2.9%), as reflected in Fig. 3.

Table 4 illustrates the distribution of serotypes across main clinical manifestations. The proportion of serotype 3 in complicated pneumonia was significantly higher than in other clinical manifestations (61; 58.1%; *p* < 0.001).

**Table 4:** Distribution of serotypes by main clinical manifestations

	3	24 F	10 A	19 A	8	23B	Others	Not typed	Total cases
Complicated pneumonia	61 (58.1%)	2 (1.9%)	1 (0.9%)	2 (1.9%)	3 (2.9%)	0 (0%)	27 (25.7%)	9 (8.6%)	105
Uncomplicated pneumonia	3 (9.7%)	4 (12.9%)	1 (3.2%)	3 (9.7%)	0 (0%)	0 (0%)	19 (61.3%)	1 (3.2%)	31
Meningitis	4 (12.5%)	4 (12.5%)	1 (3.1%)	0 (0%)	3 (9.4%)	0 (0%)	19 (59.4%)	1 (3.1%)	32
Bacteremia	0 (0%)	1 (3.4%)	3 (10.3%)	2 (6.9%)	0 (0%)	3 (10.3%)	17 (58.6%)	3 (10.3%)	29
Osteoarticular infection	1 (10.0%)	0 (0%)	2 (20.0%)	0 (0%)	0 (0%)	2 (20%)	5 (50%)	0 (0%)	10

Of the 205 episodes with an identified serotype, 93 (45.4%) were caused by a PCV13-included serotype, 72 (77.4%) of which were serotype 3. Regarding new conjugate vaccines, 97 episodes (47.3%) were caused by a PCV15-included serotype and 132 (64.4%) by a PCV20-included serotype.

Among ninety-three patients with IPD caused by PCV13-included serotype, 65 (69.9.%) were fully vaccinated. Therefore, the vaccine failure proportion observed in our study was 69.9%. Table 5 shows the distribution of serotypes in the episodes of vaccine failure (of 205 episodes with serotype). Serotype 3 was the leading serotype causing vaccine failure (53 episodes of 65; 81.5%).

**Table 5:** Distribution of serotypes in episodes of vaccine failure

Serotype	*n* (%)
3	53 (81.5%)
19 A	6 (9.2%)
14	2 (3.1%)
4	1 (1.5%)
7 FA	1 (1.5%)
9 V	1 (1.5%)
19 F	1 (1.5%)

**Fig. 4:**
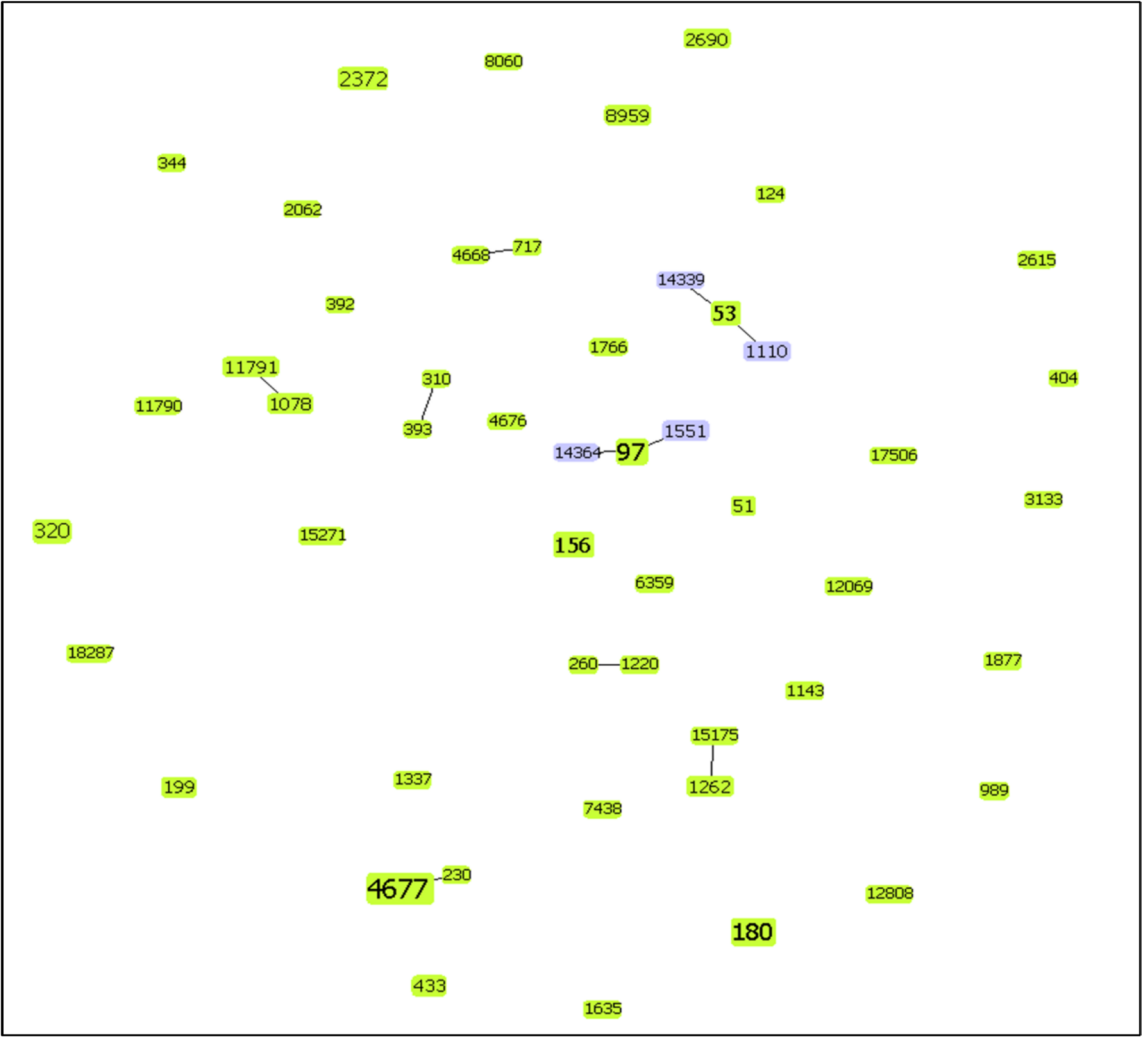
Molecular analysis by multilocus sequence typing

Molecular analysis by multilocus sequence typing was performed for 89 of 106 strains isolated by culture (84%). Fifty different ST were detected, distributed in 8 clonal complexes (CC) and 32 singletons (Fig. 4).

The major clonal complex was CC230 (ST4677 and ST230), detected in 11 strains, all expressing serotype 24 F and non-susceptible to penicillin. The second prevalent CC was CC97 (ST97, ST1551, and ST14364), detected in 9 strains, all serotype 10A and penicillin-susceptible, and the third main clonal complex was CC53 (ST53, ST1110, and ST14339) with seven strains, all serotype 8 and penicillin-susceptible. ST180 expressing serotype 3 was also prevalent with six strains all of them penicillin susceptible.

**Fig. 5:**
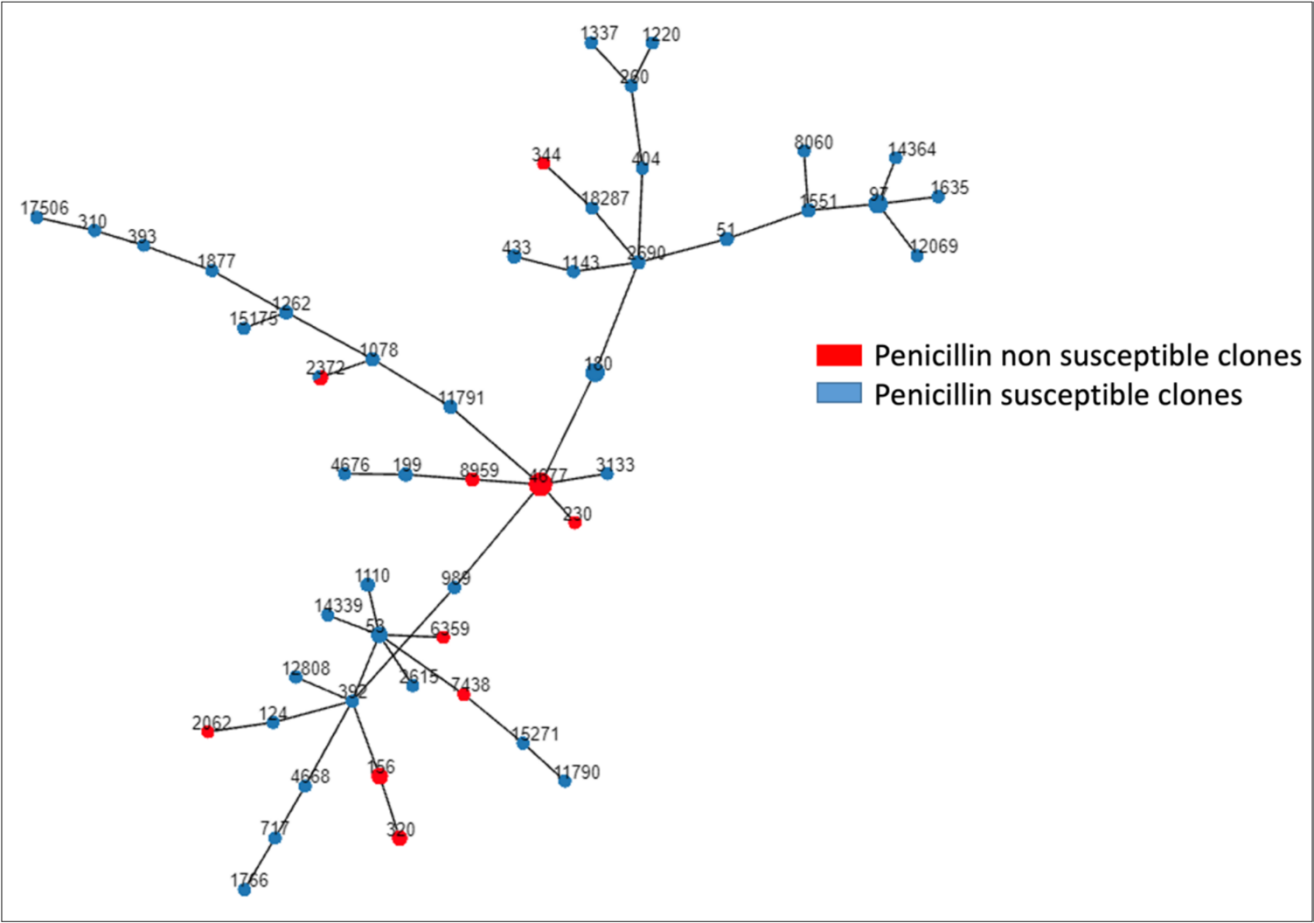
Clonal composition of the strains according to penicillin susceptibility

A slight decrease in non-susceptible clones was observed comparing the years 2018–2019 versus 2020–2021: 16 of 48 strains (33.3%) vs 2 of 15 (13.3%) (*p* = 0.07); however, in 2022, the rate of non-susceptible strains was like the first period (8 of 26 strains, 30.7%). Notably, with molecular characterization, CC230 was detected in 10 (40%) of the 25 penicillin non-susceptible strains. Figure 5 shows the clonal composition of the strains according to penicillin susceptibility.

Antibiotic sensitivity was available for 91 of 106 (85.8%) strains. The remaining tests could not be conducted because the strain was not viable, and antimicrobial studies were not available. The proportion of strains non-susceptible to penicillin or cefotaxime according to meningeal breakpoints remained stable throughout the study with non-significant differences (*p* = 0.458 for non-susceptible to penicillin and *p* = 0.278 for non-susceptible to cefotaxime) (Table 6).

**Table 6:** Evolution of non-susceptible strains throughout the study period

	Pre pandemic period 2018–2019	Early pandemic period 2020–2021	Postpandemic period 2022	Total
Number of total episodes with antimicrobial studies	48	17	26	91
Penicillin non-susceptible	16 (33.3%)	3 (17.6%)	7 (26.9%)	26 (28.5%)
Cefotaxime non-susceptible	7 (14.5%)	1 (5.8%)	1 (3.8%)	9 (9.9%)

## Discussion

Our research team has tracked IPD in Catalonia for 20 years, noting a steady decrease in incidence over time with the introduction of conjugate pneumococcal vaccines. The incidence rate of IPD after the initial implementation of the PCV13 vaccine decreased from 89.7 episodes/100.000 population in the period of 2007–2009 to 34.4 episodes/100.000 population in the period of 2012–2016, a reduction of − 62% (95% CI, − 69 to − 54%; *p* < 0.001) [31].

Our data show a continued decrease in IPD incidence. However, the incidence of IPD was much lower than expected in 2020 and 2021 because of the COVID- 19 pandemic. During those years, respiratory-transmitted diseases decreased significantly due to the use of masks, isolation measures, and social distancing [12]. Following a notable decline during the pandemic years, the incidence of IPD has rebounded to similar levels to those observed before the pandemic, a trend also reported in other studies [32, 33].

Regarding the way in which the diagnosis was obtained, it should be noted that in 51.8% of the cases, the diagnosis was made exclusively by real-time PCR. Although the European Union accepts a positive pneumococcal PCR in blood as evidence that pneumococcus is the cause of infection https://www.ecdc.europa.eu/en/all-topics/eu-case-definitions, some authors have questioned this fact, alluding to the fact that asymptomatic carriers can have a positive PCR in blood [34]. However, other studies reported a high specificity of this technique [35, 36]. In our sample, we considered that all the pneumococcal PCR in blood corresponded to actual cases of infection and not colonization, since the clinical picture was compatible in all of them: high fever, crackles or hypophones on auscultation, alveolar consolidation on chest X-ray, analytical alteration, in cases of pneumonia, high fever, and analytical alteration, in cases of bacteremia without focus.

Furthermore, the use of real-time PCR has provided a more accurate reflection of the true incidence of serotype 3 IPD, a penicillin-susceptible serotype that is underdiagnosed by culture as are other susceptible serotypes [37].

Despite the global decline in IPD rates and the high coverage of PCV13, serotype 3 remains a significant cause of severe illness among vaccinated children in Catalonia [10, 38]. It is currently the primary cause of IPD and predominantly causes complicated pneumonia in patients with no previous underlying disease. The significant impact of serotype 3 has been observed in other regions of Spain and across Europe.

Previous research has identified instances of vaccine failure related to serotype 3 [31, 39]. Evidence indicates that children who receive the PCV13 vaccine exhibit diminished antibody levels against serotype 3, which may play a role in the observed vaccine failures [40, 41]. Serotype 3 is not entirely covered by the PCV13 vaccine, leading to a significant and growing number of vaccine failures. As a result, the Catalan Health authorities decided in October 2023 to replace PCV13 with PCV15 in the systematic vaccination schedule, as PCV15 seems to offer better protection against this serotype [17, 18].

In March 2024, the European Commission authorized the use of PCV20 in children [42]. This vaccine could increase serotype coverage by 16% compared to PCV15. In our series, ten cases were caused by serotype 10 A and seven by serotype 8, covered only by PCV20. It will be interesting to see how these serotypes and serotype 3 develop in Catalonia in the upcoming years. Such data will allow the Health Authorities of Catalonia to make a more informed decision about maintaining PCV15 or transitioning to PCV20.

Genomic surveillance revealed that the main clone detected in our series was the multiresistant CC230 (GPSC10), with all strains expressing a non-vaccine serotype 24 F. We have previously reported a rapid replacement in the serotype composition of this clone from serotype 19 A to 24 F during the introduction of PCV13 in our country and after a rapid spread from Europe and other continents [7, 31]. This is a cause for concern and underscores the need to include serotype 24 F in the new conjugate vaccines.

Over the years of studying IPD, we have observed a significant rise in the proportion of patients with predisposing diseases affected by IPD: from 1.5% in 2007–2009 to 5.3% in 2012–2016 and 12.3% in 2018–2022 [7, 31]. This increase may be due to the growing number of vaccinated children, which leaves patients with predisposing diseases more vulnerable compared to healthy children. A possible explanation is that the vaccine may be less effective for them or require different vaccination regimens. Additional studies are needed to explore these cases and determine the cause of this increase.

In alignment with our previous research [7], complicated pneumonia (including pleural effusion, empyema, and lung necrosis) remains the primary clinical presentation of IPD in Catalonia and is mainly produced by serotype 3. While the prognosis is often favorable, complicated pneumonia treatment requires extended courses of antibiotics, pleural drainage occasionally, and prolonged hospitalization.

All the children were admitted to the hospital. The most prolonged median stay was in patients with sepsis. Prolonged hospital stays were due to serious complications such as acute renal failure due to C3 glomerulopathy requiring hemodialysis and immunomodulatory treatment, septic shock requiring peritoneal dialysis due to pneumococcal peritonitis, and mediastinitis requiring surgical drainage. In addition, some patients had complex underlying conditions, including a history of bone marrow transplantation, or esophageal atresia, which further prolonged recovery.

A limitation of our study is that we included only patients with microbiologically confirmed IPD, so we may be underestimating the actual burden of IPD in our population. In addition, the three hospitals are reference hospitals where the most severe diseases are treated. This is particularly relevant for cases of uncomplicated pneumonia, which may have been admitted to non-referral centers.

Another limitation is that not all samples could be serotyped. Additionally, the PCR serotyping methods sometimes lacked the precision to distinguish between two or three serotypes. However, these limitations did not have many implications for our sample’s general characterization of IPD.

To conclude, in the systematic 13-valent conjugate vaccine era, we have observed stability in IPD cases, except for the decrease during the 2 years of the pandemic. Serotype 3 is currently the most common serotype, responsible for many vaccine failures in fully vaccinated children. Serotype 3 often leads to complicated pneumonia in patients without underlying health conditions. These findings underscore the need for ongoing surveillance of IPD patterns to decide on the optimal vaccine strategy for future use.

## Data Availability

All data were treated as confidential, and records were accessed anonymously. The anonymised data collected are available as open data via the Research Electronic Data hosted at Agència de Salut Pública de Catalunya.

## Abbreviations

CC: Clonal complex
CI: Confidence interval
CSF: Cerebrospinal fluid
Ct: Cycle threshold
COVID- 19: Coronavirus disease 2019
DNA: Deoxyribonucleic acid
HIV: Human immunodeficiency virus
ICU: Intensive care unit
IPD: Invasive pneumococcal disease
MIC: Minimum inhibitory concentration
MLST: Multilocus sequence typing
PICU: Pediatric intensive care unit
PCR: Polymerase chain reaction
PCV7: Pneumococcal conjugate vaccine, 7-valent
PCV13: Pneumococcal conjugate vaccine, 13-valent
PCV15: Pneumococcal conjugate vaccine, 15-valent
PCV20: Pneumococcal conjugate vaccine, 20-valent
SD: Standard deviation
SLV: Single locus variant
ST: Sequence type
Vs: Versus
WHO: World Health Organization
